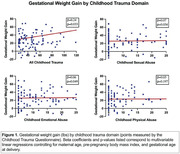# Oral Concurrent Session 7 – Diabetes: Saturday, February 1, 2025 8:00 AM – 10:15 AM

**DOI:** 10.1002/pmf2.12018

**Published:** 2025-01-05

## 77 | Does Large for Gestational Age and/or Polyhydramnios Warrant Rescreening for Gestational Diabetes Mellitus?

Henry Lesser; A. Dhanya Mackeen; David Chromey, II; Amanda J. Young; Celia Gray; Michael J. Paglia

*Geisinger Medical Center, Danville, PA*

8:00 AM ‐ 8:15 AM

**Objective**: Gestational diabetes mellitus (GDM) screening is often repeated in the 3rd trimester when large for gestational age (LGA) and/or polyhydramnios is diagnosed. We aimed to assess whether patients who did not rescreen in the 3rd trimester had any worse outcomes when compared to those that did.

**Study Design**: This is a retrospective cohort study from 1/1/11‐3/1/24 of term, singleton pregnancies diagnosed with LGA and/or polyhydramnios after a normal GDM screen at 24‐30 weeks. Patients with diabetes mellitus, fetal growth restriction, or fetal anomalies were excluded. Non‐inferiority analyses were conducted to compare outcomes of individuals who underwent rescreening with those who did not. Noninferiority was established if the lower limit of the one‐sided 95% confidence interval (CI) for the event rate in the ‘did not rescreen’ group did not exceed the relative margin of error from the event rate in the ‘did rescreen’ group. The lower bound of the 95% CI and corresponding p‐values were reported. A p‐value of < 0.05 was considered significant. 

**Results**: 1693 pregnancies were included: 1317 did not rescreen and 376 rescreened. In patients that rescreened, 13.6 % were diagnosed with GDM, of which 22% required medication. Outcomes were not worse for those who did not rescreen than those that did rescreen for birthweight (BW) (3758.8g vs 3813.6g p< 0.01), BW ≥4000 (31.9% vs. 34.1%, p< 0.01), obstetric anal sphincter injuries (3.2% vs. 2.1%, p< 0.01), preeclampsia (3.6% vs 5.3%, p< 0.01), neonatal intensive care unit admission (7.6% vs 7.4%, p< 0.01), or GDM in future pregnancies (2.3% vs. 2.4%, p< 0.01).   

**Conclusion**: Patients that did not rescreen for GDM following the diagnosis of LGA and/or polyhydramnios in the 3rd trimester did not have worse birth outcomes than those that did rescreen. This may demonstrate that despite diagnosing GDM in the 3rd trimester, there is insufficient time to intervene and change outcomes and therefore rescreening all patients with LGA and/or polyhydramnios may not be beneficial. 

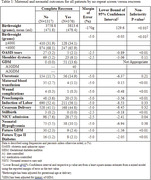

## 78 | Breastfeeding Patterns Among Parturients with Diabetes: A secondary Analysis of the MOMPOD Randomized Clinical Trial

Minhazur R. Sarker^1^; Marni B. Jacobs^2^; Kim Boggess^3^; Ashley N. Battarbee^4^; Gladys (Sandy) A. Ramos^1^

*^1^University of California, San Diego, San Diego, CA; ^2^University of California, San Diego Health, San Diego, CA; ^3^University of North Carolina, Chapel Hill, NC; ^4^Center for Women's Reproductive Health, University of Alabama at Birmingham, Birmingham, AL*

8:15 AM ‐ 8:30 AM

**Objective**: Insulin resistance is associated with decreased milk supply in those lactating. Metformin is hypothesized to increase breast milk production by presumptively improving insulin resistance, suggesting use in pregnancy may increase breastfeeding (BF) success. We aimed to determine the association between metformin use for treatment of preexisting type 2 diabetes mellitus (T2DM) or DM diagnosed in early pregnancy (early DM) and BF patterns.

**Study Design**: This was a planned secondary analysis of the MOMPOD randomized controlled trial of metformin versus placebo in insulin treated T2DM or early DM. We included parturients who delivered a living neonate, received at least one dose of study treatment, endorsed an intention to BF, and completed a BF survey. BF intentions and BF outcomes were collected utilizing a BF questionnaire at 24‐30 weeks and 30 days postpartum (PP) respectively. The primary outcome was immediate BF, defined as any BF on PP day 1‐3. Secondary outcomes included time to milk production, exclusive or partial BF at 30 days PP, breast size and issues with BF. Baseline characteristics and outcomes were compared using either chi‐square, t‐test, or Wilcoxon tests, as appropriate.

**Results**: Among the 794 women randomized and receiving medication in the primary trial, 378 (47.6%) met inclusion criteria with 194 (51.3%) in metformin and 184 (49.7%) in placebo groups. There were no significant differences in baseline demographics (Table 1). Immediate BF was comparable between groups (91.1% vs 88.9%, p = 0.53) and there was no difference in time to milk production (Table 2). Thirty days PP, BF was lower in both groups but there was no difference between metformin and placebo groups (76.0% vs 66.7%, p = 0.11). There were also no differences in partial or exclusive BF, in breast cup or bra size, or issues with BF (Table 2).

**Conclusion**: Our data suggest no association between metformin use and BF patterns in those with T2DM or early DM. Antepartum metformin should not be recommended to improve BF success. Further studies should focus on methods to improve sustained BF in this population.

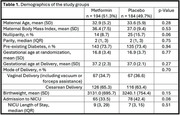

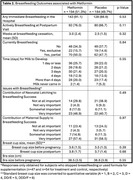

## 79 | Predicting Future Diabetes: Impact of Abnormal Pregnancy OGTT Patterns

Yael Winter Shafran^1^; Tal Schiller^2^; Alena Kirzhner^2^; Edi Vaisbuch^2^

*^1^Kaplan Medical Center and REALIFE Research Group, Rehovot, HaMerkaz; ^2^Kaplan Medical Center, Rehovot, HaMerkaz*

8:30 AM ‐ 8:45 AM

**Objective**: Evidence suggests that glucose challenge tests during pregnancy can predict long‐term metabolic abnormalities. We evaluated the predictive value of Oral Glucose Tolerance Test (OGTT) patterns during pregnancy for future diabetes mellitus (DM) development.

**Study Design**: This retrospective cohort study included pregnant people who performed a 100‐gram OGTT. Those with pre‐existing diabetes, recent diabetic medication or steroid use, and incomplete OGTT results were excluded. The predictive value of the OGTT for future DM was assessed using a large healthcare system database, with a follow‐up of at least 8 years. Two models were analyzed, one comparing two or more abnormal results to less than two abnormal results in OGTT and the second analyzing the predictive value by the number of abnormal results and the “location” of a single abnormal result (i.e. fasting, one‐hour, two‐hour, and three‐hour post glucose ingestion).

**Results**: Of the 10,372 individuals identified, 8,157 met the inclusion criteria. Those with a pathological OGTT were older, more obese, and had higher rates of hypertension and dyslipidemia. During follow‐up, 22.1% of people with ^3^2 abnormal results developed DM compared to only 3.8% in the group with < 2 abnormal results (HR 5.22, 95% CI 4.33‐6.30). Compared to those with all four results normal, the hazard ratio (HR) for developing DM with one, two, three, or four abnormal results were 3.45 (95% CI 2.77‐4.53), 6.55 (95% CI 5.12‐8.36), 10.26 (95% CI 7.54‐13.96), and 18.63 (95% CI 11.87‐29.25), respectively. Among people with only one abnormal result, abnormal fasting glucose showed the highest risk (HR 4.01, 95% CI 2.7‐5.92).

**Conclusion**: This study demonstrates that even one abnormal result (with the fasting glucose posing the highest risk) in the 100‐gram OGTT is a risk factor for the future development of DM. The risk further increases with each additional abnormal result. These findings underscore the importance of postnatal follow‐up and early modifiable interventions even for individuals with one abnormal OGTT result to potentially mitigate future DM risks.

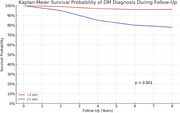

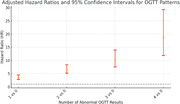

## 80 | Effect of Breastfeeding on the Early Postpartum Lipid Profile in Women with Gestational Diabetes Mellitus

Harumi Kanzawa^1^; Hiroshi Yamashita^1^; Ichiro Yasuhi^2^

*^1^NHO Nagasaki Medical Center, Omura City, Nagasaki; ^2^NHO Nagasaki Medical Center, Omura‐City, Nagasaki*

8:45 AM ‐ 9:00 AM

**Objective**: Women with a history of gestational diabetes mellitus (GDM) are at high risk for developing type 2 diabetes and metabolic syndrome. Although breastfeeding is recognized as effective in preventing these diseases, its effect on postpartum lipid metabolism remains unclear. This study aimed to investigate the association between breastfeeding and early postpartum lipid profiles in Japanese women with GDM.

**Study Design**: This prospective cohort study included singleton pregnant women with GDM enrolled during pregnancy. An oral glucose tolerance test (OGTT) was conducted at 6 to 9 weeks postpartum. Fasting lipid profiles, including total cholesterol (T‐cho), triglycerides (TG), high‐density lipoprotein (HDL), and low‐density lipoprotein cholesterol (LDL‐cho) levels, were measured. Breastfeeding intensity was categorized into six levels, from exclusive breastfeeding (Level 1) to formula feeding only (Level 6). Breastfeeding of 80% or more (Levels 1‐3) was defined as high‐intensity breastfeeding (HIB). Adjusted odds ratios (aOR) of hypertriglyceridemia (≥150 mg/dL) and hyper LDL cholesterolemia (≥140 mg/dL) were calculated.

**Results**: A total of 501 participants underwent a OGTT at 7.3 weeks postpartum. Lipid levels were T‐cho 210 ± 33 mg/dL, TG 73 ± 49 mg/dL, HDL 72 ± 17 mg/dL, and LDL 127 ± 30 mg/dL. Breastfeeding intensity levels were 55%, 14%, 9%, 10%, 10%, and 3% from Levels 1 to 6, respectively, resulting in 77% for HIB. All lipids, except T‐cho, were significantly associated with breastfeeding intensity (**Table**). Compared to women with non‐HIB, those who engaged in HIB showed significantly lower prevalence of hypertriglyceridemia and hyper LDL cholesterolemia, with aORs of 0.21 (95% CI: 0.11‐0.40) and 0.48 (0.31‐0.76), respectively.

**Conclusion**: Breastfeeding significantly improved the early postpartum lipid profiles of women with GDM as breastfeeding intensity increased.

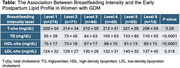

## 81 | Rates of Risk Appropriate Postpartum Care within Six Months after Delivery

Jennifer F. Culhane; Anna Denoble; Olivia Paoletti; Caitlin Partridge; Lisbet S. Lundsberg

*Yale School of Medicine, New Haven, CT*

9:00 AM ‐ 9:15 AM

**Objective**: Pregnancy is a “stress test”, providing a window into future health risk. Enthusiasm for postpartum (PP) care (PPC) to mitigate maternal mortality and long‐term adverse health outcomes is growing. We aimed to evaluate domain‐appropriate 6‐month PPC utilization by maternal antenatal risk factors.

**Study Design**: This was a retrospective study of delivery admissions occurring at a health system from 2013‐2023 with evidence of ≥1 prenatal care visit. All encounters from delivery through 6‐months PP were identified. Iterative review of PP encounters among random samples of 10,000 patients allowed selection of ICD‐10 codes associated with PPC and 11 condition‐based care domains. PPC rates were calculated by 1) any encounter, 2) traditional PPC (Z39 or V24), and 3) domain‐specific PPC (Figure). Patients with risk factors (chronic hypertension (cHTN), hypertensive disorders of pregnancy, type 1 or 2 diabetes, gestational diabetes, depression/anxiety, and substance use disorder (SUD)) were identified based on ICD‐10 codes. Rates of domain‐appropriate PPC by risk factors were compared using Chi‐square tests.

**Results**: N = 60,933 were included. 90% of patients had any encounter within 6 months PP and 78% had a traditional PPC visit (Figure). Across specific domains, 6‐month PPC rates ranged from 0.6% for problems with social determinants of health (e.g. inadequate housing, lack of adequate foods, extreme poverty) to a high of 29.0% for contraception care. Exploring rates of domain‐specific PPC by maternal risk factors, cHTN had the highest rate of appropriate PPC follow‐up (54.5%), while patients with SUD had the lowest (16.6%) (Table). With the exception of cHTN, < 50% of patients with antenatal risk factors obtained domain appropriate PPC within 6 months of delivery.

**Conclusion**: Although we observed high rates of PPC overall, care specific to maternal risk factors was lacking, with rates mostly under 50%. Care for depression/anxiety and SUD treatment was particularly low. Major improvements are needed if PPC is to be a target to combat increasing rates of maternal mortality and improve health over the life course.

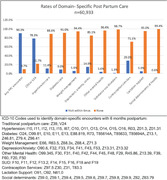

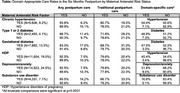

## 82 | Metformin Use in Pregnancy Decreases Neonatal Fat Free Mass

Claire E. Jensen^1^; Ashley N. Battarbee^2^; Kim Boggess^1^; Kjersti M. Aagaard^3^; On behalf of the MOMPOD Consortium

*^1^University of North Carolina, Chapel Hill, NC; ^2^Center for Women's Reproductive Health, University of Alabama at Birmingham, Birmingham, AL; ^3^Boston Children's Hospital, Division of Fetal Medicine and Surgery, Boston, MA; HCA Healthcare and HCA Healthcare Research Institute, Nashville, TN; HCA Texas Maternal Fetal Medicine, Houston, TX; Baylor College of Medicine and Texas Children's Hospital, Houston, TX, Boston, MA*

9:15 AM ‐ 9:30 AM

**Objective**: Murine and primate models treated with maternal metformin exhibit fetal bioaccumulation, leading to reduced muscle and retroperitoneal fat mass with fetal growth restriction. In clinical trials, maternal metformin use during pregnancy results in smaller neonates with reduced LGA risk, alongside a variable increased risk for SGA births. We hypothesized that the observed decreased prevalence of LGA births with maternal metformin use during pregnancy results from reduced accumulation of fetal fat free mass (FFM, i.e., muscle accretion) rather than diminished adiposity.

**Study Design**: Secondary analysis of a large multicenter RCT (MOMPOD) of metformin 1000mg BID vs. placebo in singleton pregnancies at 11‐23 weeks with insulin‐treated early diabetes or pre‐existing T2DM. We included all participants who took ≥ 1 dose of study drug and had neonatal anthropometry. Multivariable linear regression estimated the association between metformin use and neonatal FFM and evaluated for effect modification by key covariates.

**Results**: Of n = 794 participants, 437 (55%) completed comprehensive neonatal anthropometry. They were demographically similar to the full cohort and balanced between treatment groups. Mean FFM was 2786 g (SD 408.15g, 86.2% of birth weight). FFM was 81.8g less in neonates exposed to metformin (95% CI ‐157.59, ‐4.56). The association between metformin exposure and FFM did not vary by neonatal sex, maternal race/ethnicity, pregestational diabetes diagnosis, nor excess maternal weight gain (Table 1). There was a trend toward effect modification by duration of exposure (Figure 1), indicating that the metformin‐associated decline in muscle accretion increased with gestational age (p = 0.21).

**Conclusion**: Metformin is associated with lower neonatal muscle accretion, measured as FFM. Our finding of decreased FFM among metformin‐exposed neonates adds to the mounting evidence raising concern for transplacental transfer and bioaccumulation in the fetus, which may restrict healthy physiologic growth patterns and potentially increase risk for adverse long‐term cardiometabolic outcomes.

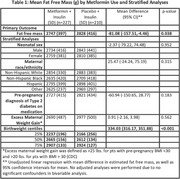

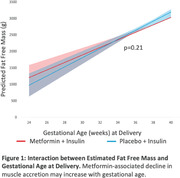

## 83 | Interaction between Metformin and Baseline Insulin Requirements on Neonatal Outcomes in Pregnancies with Type‐2 Diabetes

Kevin S. Shrestha^1^; Claire E. Jensen^2^; Kim Boggess^2^; Gladys (Sandy) A. Ramos^3^; Ashley N. Battarbee^4^

*^1^University of Alabama at Birmingham, Birmingham, AL; ^2^University of North Carolina, Chapel Hill, NC; ^3^University of California, San Diego, San Diego, CA; ^4^Center for Women's Reproductive Health, University of Alabama at Birmingham, Birmingham, AL*

9:30 AM ‐ 9:45 AM

**Objective**: Metformin did not improve composite neonatal morbidity among insulin‐treated preexisting type 2 diabetes (T2D) in two recent RCTs. Although metformin reduced insulin requirements in the MiTy trial, there was no difference observed in the MOMPOD trial. Given trial heterogeneity, we aimed to determine if baseline insulin requirements modified the effect of metformin on neonatal outcomes.

**Study Design**: Secondary analysis of the MOMPOD trial, an RCT of metformin versus placebo in insulin‐treated T2D or diabetes diagnosed in early pregnancy. Our primary outcome was the composite of neonatal complications from the parent trial (Table). Total daily dose (TDD) of insulin at randomization was evaluated as an effect modifier of the relationship between metformin and the primary outcome using a likelihood ratio test with p< 0.10 as significant. To further investigate effect modification, we evaluated the association between metformin and outcomes among subgroups with TDD < 30U, >60U, and >90U using multivariable Poisson regression with robust error variance and ordinary least squares regression.

**Results**: Of the 794 participants included, the median baseline TDD was 62 units (IQR 35, 88). There was significant interaction between metformin and TDD in relation to the primary outcome (Figure). In subgroup analyses of TDD< 30, metformin was associated with lower risk of composite neonatal outcome, LGA, neonatal fat mass, preterm birth, and NICU admission (Table). However, there were no differences in outcomes with metformin vs placebo among participants with TDD >60, with exception for smaller increase in insulin TDD during pregnancy (Table). Findings were similar for participants with TDD >90U.

**Conclusion**: The effect of metformin on neonatal outcomes in the MOMPOD trial cohort differed by baseline insulin requirements. Contrary to our hypothesis, metformin was associated with fewer adverse neonatal outcomes among those with low, not high, TDD. Further studies are needed to confirm our findings and evaluate the reason why metformin is beneficial for pregnancies with low insulin requirements.

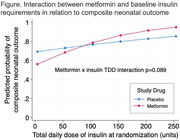

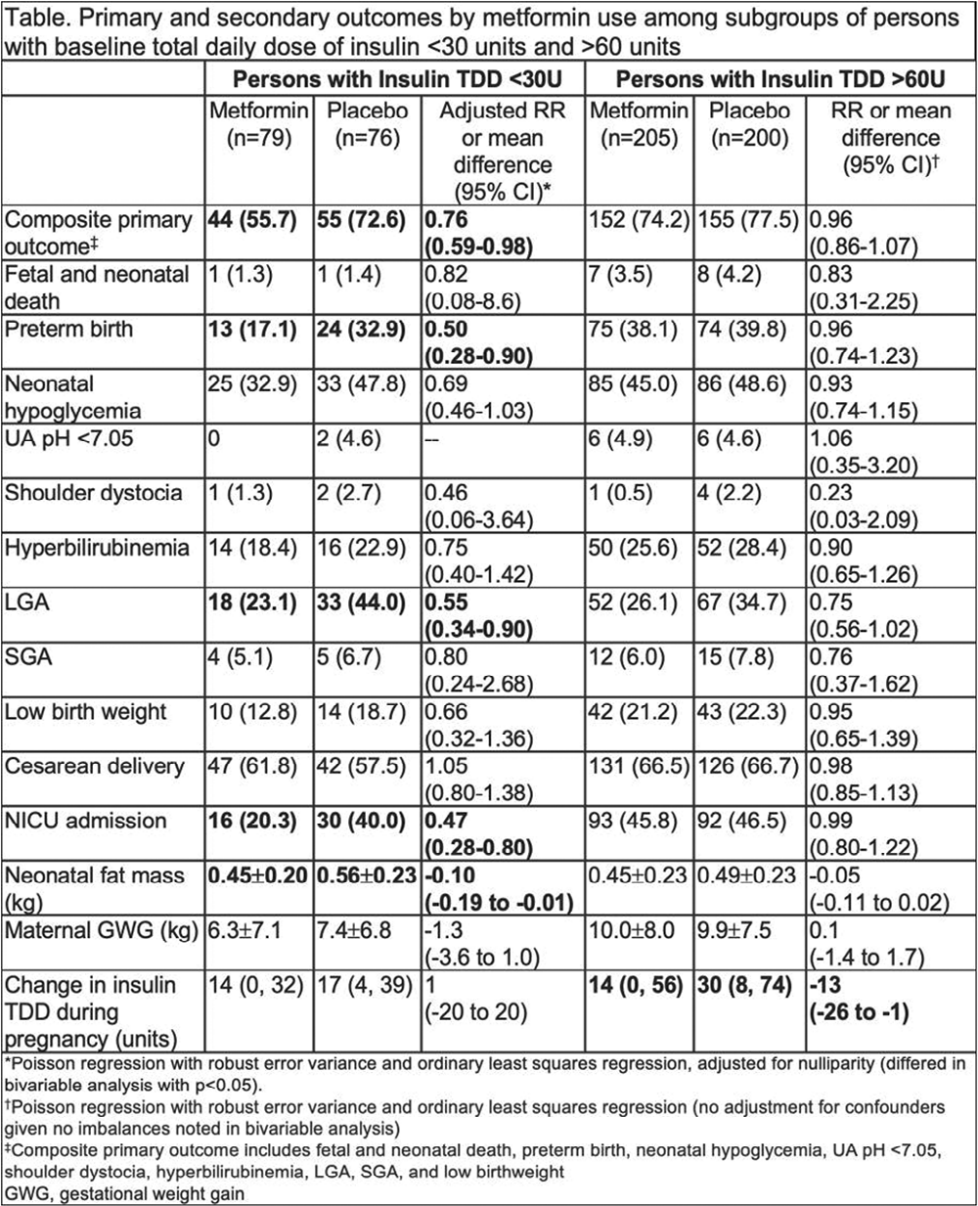

## 84 | The Placental Transcriptome in Pregnancies Complicated by A2 Gestational Diabetes Mellitus

Morgan E. Wasickanin^1^; Samantha Carson^1^; Hillary Kinsman^1^; Lydia Bettridge^1^; Katherine E. Free^1^; Jennifer Damicis^1^; Emily Sheikh^1^; Robert Walton^1^; Peter Napolitano^2^; Nicholas Ieronimakis^1^

*^1^Madigan Army Medical Center, Tacoma, WA; ^2^University of Washington, Seattle, WA*

9:45 AM ‐ 10:00 AM

**Objective**: Adverse obstetric and fetal outcomes are increased in pregnancies complicated with A2 gestational diabetes mellitus (A2GDM) compared to non‐diabetic pregnancies. Placental dysfunction is thought to be attributed to some of the complications in A2GDM, yet the underlying molecular mechanisms remain unknown. We hypothesized that transcriptional differences in human placentas from A2GDM pregnancies may relate to tissue dysfunction. To test our hypothesis, we compared the transcriptome within placentas delivered from A2GDM versus uncomplicated pregnancies.

**Study Design**: Intervillous biopsies were dissected from unlabored placentas delivered by cesarean. The extracted RNA was depleted for both adult and fetal globin transcripts prior to transcriptional analysis by mRNA‐seq (n = 10/condition). Alignment and differential gene expression (DEGs) analysis was conducted using Illumina's Dragen software. DEGs were considered statistically significant based on a cut‐off adjusted p‐value of < 0.05 using Benajamini‐Hochberg false discovery rate correction.

**Results**: Between A2GDM versus uncomplicated placentas 14,825 DEGs were identified. Among these 1092 had a p < 0.05. When p‐values were adjusted, the following 3 DEGs were significantly upregulated with A2GDM: AC068547.1, MMRN1, and ZNF780A (Figure 1).

**Conclusion**: The intervillous transcriptome in placentas from pregnancies complicated by A2GDM appears different than those from uncomplicated pregnancies. AC068547.1, a gene that codes for the calcium voltage‐gated channel auxiliary subunit beta 4 (CACNB4) protein, was upregulated in a subset of placentas from A2GDM pregnancies. MMRN1 and ZF780A, proteins that function in coagulation and transcription regulation respectively, were more consistently upregulated in A2GDM samples. Further investigation is warranted to understand the role of these factors in placentas complicated by gestational diabetes mellitus.

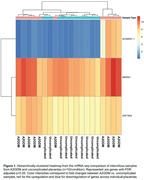

## 85 | Childhood Sexual and Emotional Maltreatment are Associated with Increased Gestational Weight Gain

NATALIE E. POLIEKTOV^1^; Mariana Rocha^1^; Kaitlyn Stanhope^2^; Lauren Holt^1^; Alicia Smith^1^; Vasiliki Michopoulos^1^; Suchitra Chandrasekaran^3^

*^1^Emory University School of Medicine, Atlanta, GA; ^2^Rollins School of Public Health, Atlanta, GA; ^3^Emory University, Atlanta, GA*

10:00 AM ‐ 10:15 AM

**Objective**: Gestational weight gain (GWG) is associated with adverse metabolic outcomes (AMO) for a mother & infant. Prior research suggests an association between maternal childhood trauma (CT) and increased GWG; however, little is known about how different domains of CT (e.g., sexual abuse (SA), emotional abuse (EA) & physical abuse (PA)) influence GWG. We aimed to study the association between CT & its domains with GWG.

**Study Design**: This is a prospective longitudinal cohort study investigating associations between trauma exposure & pregnancy outcomes at an urban safety net hospital. CT was assessed using the Childhood Trauma Questionnaire (CTQ), a validated measure of 5 trauma domains, as a continuous score & dichotomized as CT (CTQ score >/ =  35) or no CT (CTQ score < 35). Adult trauma was assessed using the Traumatic Experiences Inventory. Weight & body mass index (BMI) were recorded during each trimester. Excessive GWG (EGWG) was defined as GWG exceeding BMI‐based recommendations from ACOG. Multivariable linear regression and logistic regression were performed controlling for maternal age, pre‐pregnancy BMI, & gestational age at delivery.

**Results**: N = 335 subjects were enrolled in the study. CTQ total, SA, & EA scores were positively correlated with increased GWG *(b = 0.24, 95% CI 0.02‐0.46, p = 0.033; b = 0.07, 95% CI 0.01‐0.1, p = 0.034; b = 0.06, 95% CI 0.00‐0.12, p = 0.049, respectively)* (Figure 1). Mean GWG was 9 pounds (lb) higher in those with SA vs. no SA *(30.5 lb vs. 21.5 lb)* whereas mean GWG was 4 lb higher in those with EA vs. no EA *(27.5 lb vs. 23.4 lb)*. Further, there was 1.5‐fold higher odds of having EGWG with CT vs. no CT *(OR 1.47, 95% CI 0.94–2.33, p = 0.092)*. Childhood PA & adult trauma were not associated with GWG.

**Conclusion**: Using detailed trauma exposure measures, our data indicate that childhood SA & EA, but not PA or adult trauma, are associated with increased GWG during pregnancy. Further research is needed to understand the complex interactions between specific domains of trauma exposure & AMO in order to improve maternal & infant health.